# Supplementary material for: Managerial overconfidence in capital structure decisions and its link to aggregate demand: An agent-based model perspective
Source: PLoS One. 2021 Aug 19;16(8):e0255537. doi: 10.1371/journal.pone.0255537 (PMC8376048; doi:10.1371/journal.pone.0255537)
Supplement: S1 Table — (DOCX) [file pone.0255537.s001.docx]

S1 Table: Parameter values.

| **Parameter** | **Value** | **Description** |
| --- | --- | --- |
| $Nf$ | 500 | Number of C-firms |
| $\lambda_{yd_{i}^{e}}$ | 0.25 | Expectation parameter (1) |
| $\lambda_{g}$ | 0.2 | Growth weight for expected demand (1) |
| $\theta_{inv}$ | 0.02 | Firms desired inventory to expected demand ratio (2) |
| $\lambda_{inv}$ | 0.5 | Adjustment speed of supplementary output (3) |
| $\nu$ | 12 | Capital to output ratio (6) |
| $\alpha$ | 0.0025 | Labour productivity growth (7) |
| $\mu_{0}$ | 0.24 | Autonomous mark-ups on unit cost (9) |
| $\mu_{1}$ | 0.15 | Utilization impact on mark-up on unit cost (9) |
| $\mu_{2}$ | 0.2 | Market share impact on mark up on unit cost (9) |
| $\lambda_{p}^{0}$ | 0.05 | Price update probability (10) |
| $\lambda_{p}^{1}$ | 0.5 | Price update probability (10) |
| $\lambda_{p}$ | 0.5 | Adjustment speed of price (11) |
| $\mu_{\pi}$ | 0.03 | Average bias for overconfidence (13) |
| $\epsilon_{\pi}$ | 0.1 | SD for overconfidence (13) |
| $uT$ | 0.6 | Threshold utilization rate (15) |
| $\lambda_{i_{des}}^{0}$ | 0.55 | Autonomous investment decision (15) |
| $\lambda_{i_{des}}^{1}$ | 0.4 | Utilization impact on investment (15) |
| $\lambda_{d,min}$ | 0.1 | Minimum ratio of deposits (18) |
| $\lambda_{d,max}$ | 0.5 | Maximum ratio of deposits (19) |
| $\gamma_{arb}$ | 3 | Weight for the arbitrage function (24) |
| $\lambda_{ext}^{0}$ | 0.1 | Autonomous external fund decision (26) |
| $\lambda_{ext}^{1}$ | 0.1 | Relative cost impacts on external fund decision (26) |
| $\delta$ | 0.015 | Depreciation rate of capital (27) |
| $sf$ | 0.84 | Firms’ retention ratio (29) |
| $\lambda_{arb}$ | 0.1 | Adjustment speed for capital structure (30) |
| $\lambda_{f}$ | 0.3 | Adjustment speed demand distribution (37) |
| $\mu_{fEq}$ | 0 | Mean of random distribution of demand (37) |
| $\epsilon_{fEq}$ | 0.08 | SD of random distribution of demand (37) |
| $\lambda_{f}^{p}$ | 0.667 | Weight on demand distribution (38) |
| $\gamma_{fp}$ | 10 | Price generalized logistic functions for demand (39) |
| $\gamma_{fk}$ | 5 | Capital generalized logistic functions for demand (39) |
| $\nu_{fp}$ | 2 | Price generalized logistic functions for demand (39) |
| $\nu_{fk}$ | 0.5 | Capital generalized logistic functions for demand (39) |
| $\iota$ | 0.95 | Discount on scrapped capital (44) |
| $\mu_{k}$ | 1.3 | Mark-up for capital goods price (52) |
| $\omega_{0}$ | -0.088 | Phillips curve – autonomous parameter (57) |
| $\omega_{1}$ | 0.1 | Phillips curve – employment parameter (57) |
| $\omega_{2}$ | 0 | Phillips curve – inflation parameter (57) |
| $c0$ | 0.9 | Propensity to consume out of wages (61) |
| $c1$ | 0.2 | Propensity to consume out of financial incomes (61) |
| $c2$ | 0.015 | Propensity to consume out of wealth (61) |
| $\sigma_{0}^{T}$ | 1.3 | Portfolio of households (68) |
| $\sigma_{1}^{T}$ | 5 | Portfolio of households (68) |
| $\lambda_{\sigma}$ | 0.2 | Adjustment speed of households portfolio (71) |
| $\lambda_{f_{eq}}^{ret}$ | 0.7 | Weight on equity demand distribution (72) |
| $\gamma_{fEqret}$ | 6 | ROE generalized logistic functions for equity demand (72) |
| $\gamma_{fEqnw}$ | 4 | Net worth generalized logistic functions for equity demand (72) |
| $\nu_{fEqret}$ | 0.8 | ROE generalized logistic functions for equity demand (72) |
| $\nu_{fEqnw}$ | 1 | Net worth logistic functions for equity demand (72) |
| $\lambda_{feq}$ | 0.4 | Adjustment speed equity demand distribution (76) |
| $\mu_{fEq}$ | 0 | Mean of random distribution of equity demand (76) |
| $\epsilon_{fEq}$ | 0.1 | SD of random distribution of equity demand (76) |
| $\lambda_{l}$ | 0.25 | Adjustment speed lending rate (78) |
| $i_{CB}$ | 0.0015 | Central bank interest rate (79) |
| $\gamma$ | 0.075 | Risk parameter in interest rate determination (79) |
| $i_{lb,0}$ | 0.009 | Banks’ component for interest rates (80) |
| $i_{lb,1}$ | 0.05 | Banks’ component for interest rates (80) |
| $id_{0}$ | 0 | Exogenous mark-ups for deposit rate (81) |
| $\lambda_{id}$ | 0.2 | Adjustment speed deposit rate (81) |
| $\zeta_{b}$ | 0.1 | Capital adequacy ratio (82) |
| $s_{b,max}$ | 0.5 | Maximum retained earnings of banks (85) |
| $\beta$ | 0.05 | Banks’ holding of bonds (87) |
| $G_{0}$ | 0.18 | Procyclical government expenditures (89) |
| $G_{1}$ | 0.75 | Countercyclical government expenditures (89) |
| $\theta_{y}$ | 0.2 | Tax rate on output (90) |
| $\theta_{div}$ | 0.15 | Tax rate on dividends (90) |
| $\theta_{w}$ | 0.4 | Tax rate on wages (90) |
| $\theta_{k}$ | 0 | Tax rate on investment (90) |
| $\tau_{G}$ | 0.3 | Public employment share (91) |
| $i_{b,0}$ | 0.00625 | Exogenous mark-up for public bonds (93) |
| $\lambda_{ib}$ | 0.2 | Adjustment speed bond rate (93) |
| $\epsilon_{\mathrm{overconfident}}$ | 0.6 | Probability of being overconfident |
